# A Framework for Analyzing Neighbor Discovery Protocols under Non-Ideal Conditions

**DOI:** 10.3390/s21206822

**Published:** 2021-10-14

**Authors:** Jose Jaime Camacho-Escoto, Eduardo Lopez-Bolaños, Oscar Arana, Javier Gomez

**Affiliations:** 1 Telecommunications Deptartment, Engineering Faculty, National Autonomous University of Mexico (UNAM), Ciudad Universitaria, Coyoacán, Mexico City 04510, Mexico; jcamachoe@comunidad.unam.mx; 2 Department of Engineering and Computational Systems, IIMAS, National Autonomous University of Mexico, Mexico City 04510, Mexico; edlobo@comunidad.unam.mx (E.L.-B.); arana.h.oscar@comunidad.unam.mx (O.A.)

**Keywords:** neighbor discovery, reliability, sensors, IoT

## Abstract

Neighbor Discovery (ND) protocols are crucial to achieving the paradigm of interconnecting thousands of small nodes (sensors or things) to the Internet, also known as the IoT. These protocols usually assume that nodes operate with few energy resources. Therefore, they cannot be fully active all the time. The vast majority of these protocols focus on increasing the probability that two nodes become active simultaneously, thus enabling mutual discovery. In addition, these protocols assume that successful discovery is guaranteed once two nodes are simultaneously active, with very few exceptions. However, many problems can disrupt the discovery, such as channel errors, collisions, synchronization mismatches, energy availability, and so forth. Most ND protocols did not consider these factors, making them vulnerable to severe performance degradation when transmission errors occur. This paper proposes a new framework to evaluate the performance of deterministic neighbor discovery protocols when transmission errors are present. The proposed framework facilitates obtaining an analytical CDF of the discovery time of such protocols with transmissions errors without having to implement the protocol in a simulator, since is time-consuming and prone to implementation errors. We applied the framework to analyze the effect of transmission errors on the discovery time in four of the most representative ND protocols in the literature. Finally, we validate the framework accuracy for the selected protocols using extensive simulations. The results show that the CDF of discovery times provided by the framework closely matches the performance results obtained through simulating these protocols. In general, neighbor discovery protocols are deeply affected as a result of transmission errors.

## 1. Introduction

Recently, the Internet of Things (IoT) has continued attracting significant interest from academia and industry. In this new paradigm, the Internet devices are predominantly sensors and actuators that work jointly to achieve automation, maintenance, and operational control within systems like vehicles, homes, stores, industry and agriculture, among many other systems. A large portion of the IoT devices are wireless-enabled to operate without a fixed infrastructure. Furthermore, devices in such networks run on batteries or rely on intermittently available energy-harvesting sources. Thus, energy spent on communication needs to be as low as possible.

Energy-constrained devices limit their power consumption by powering down part of their peripherals and clocks to save energy during a period referred to as ‘sleep mode’. This technique has been widely used in MAC protocols since idle listening is one of the major sources of energy waste in communications [1]. A popular approach to decrease energy consumption involves duty-cycling so that nodes use the radio channel for short periods and remain in sleep mode the rest of the time. When the clocks of all participant nodes are synchronized, the duty-cycling approach enables communication, especially when their wake-up schedules are known to all the devices in the network. However, duty-cycled communication schemes under asynchronous communication remain a challenging problem. Moreover, establishing the first contact with neighbor nodes in a wireless network is one of the most important asynchronous procedures since most upper layer applications (e.g., routing or data transfer) rely on achieving an accurate neighbor node identification. This process is known as neighbor discovery (ND). The ND problem consists of efficiently establishing a first contact with neighbor devices in terms of metrics such as discovery latency, missing probability, and power consumption.

Currently, ND algorithms can be categorized into either deterministic or stochastic groups. The deterministic protocols establish a wakeup pattern to schedule periodical operations when performing the discovery of their neighbors. Deterministic protocols, in particular can be further classified into quorum-based [2,3,4,5,6], prime number-based [7,8,9], as well as dynamic listening slots [10,11], and fixed listening slots [12,13]. In contrast, stochastic schemes [14,15,16,17,18] allow nodes to transmit beacons, listen for beacons from other nodes, or sleep in a slot based on a probability distribution. Energy efficiency is ensured by choosing a lower probability for beacon transmission or for listening. On average, stochastic schemes perform better than deterministic schemes. However, stochastic schemes provide no bound on the worst-case latency, leading to longer delays, especially for the last fraction of nodes.

Most existing ND protocols focus on achieving the lowest discovery latency or reducing power consumption. However, few works take into consideration the impact of unreliable wireless links in the ND process. For instance, factors such as intermittent energy sources, random channel errors, collisions, and clock synchronization mismatches can lead to a failure of the ND process. Moreover, since most of the proposed ND protocols present metrics on ideal conditions, the performance evaluation of different ND protocols have often been very subjective, commonly ignoring their performance under non-ideal conditions. Consequently, this paper proposes a new framework to evaluate the performance of deterministic ND protocols when transmission errors are present. The proposed framework facilitates obtaining an analytical CDF of the discovery time of such protocols with transmissions errors without implementing the protocol in a simulator, which is time-consuming and prone to implementation errors. To illustrate how the framework is used, we applied it to four of the most representative ND protocols in the literature. The work in [19] is the closest to ours, yet there are three main differences. First, the main focus of this paper is to present a framework that can study the impact of transmission errors on any deterministic ND protocol. Secondly, we study the most representative ND algorithms instead of only Quorum-based algorithms. Finally, the analysis technique presented in this paper considers standard metrics such as Cumulative Distribution Functions (CDFs) of the discovery latency. Moreover, the main contribution of this study is twofold: (i) present a framework to approximate the CDF of the discovery latency of ND protocols under non-ideal conditions, and (ii) it applies the framework to four of the most representative ND protocols with their respective CDFs when transmission errors are present.

The rest of this paper is organized as follows: For clarity, Section 2 provides an overview of the most representative ND protocols and presents works carried out under non-ideal operating conditions. Section 3 introduces the proposed framework methodology to model the behavior of deterministic ND protocols under non-ideal operating conditions. Section 4 validates the framework and describes the simulations that were carried out as well as the obtained results. Section 6 discusses the main findings and Section 7 concludes the paper.

## 2. Related Work

Over the past years, the development of ND protocols has focused on strategies that achieve the discovery of neighbor nodes constrained to a bounded latency. More recently, however, researchers have focused on accomplishing ND under energy harvesting scenarios [20]. In either case, few researchers have taken into consideration the ND problem under non-ideal wireless channel conditions [19,21]. This section first introduces the most representative ND protocols. Secondly, it presents a brief description of related works and their main differences.

As aforementioned, ND protocols can be classified into two categories: deterministic or stochastic. Table 1 presents a chronological summary of the most significant ND protocols. Most ND protocols in each subclassification share a similar working principle. For instance, Quorum-based schemes [2,3,4,5,6] guarantee that two nodes have at least one activity slot in common by being active in $\sqrt{N}$ in a period of $N^{2}$ slots. These schemes result in relatively high duty cycles. Prime number-based schemes, in contrast, require a node to choose a single prime number (e.g., U-Connect [7]) or a pair of prime numbers (e.g., Disco [8]) to derive their duty cycles. A node’s activity slots will be the multiples of the selected prime number(s). In this case, discovery latency is equal to the time slot corresponding to the product of the prime numbers used by the two nodes. An extension to this approach is presented in [9]; this work uses differential codes built from each pair of relative prime numbers to carry out the ND process. Active slots can be further divided into listening and transmitting slots. For instance, in dynamic listening slots schemes, ND protocols set transmission slots at fixed positions (i.e., either at the beginning or end of a predefined cycle), and listening slots are dynamically shifted to either the left or right of successive cycles. Searchlight [10] illustrates such an approach. It uses one fixed slot at the beginning of each cycle, and a dynamic slot is shifted to the right on each consecutive cycle. Another example is Blinddate [11], that uses one static slot in each cycle and two dynamic listening slots, one shifted to the right and one to the left in each consecutive cycle. The last classification considered consists of a fixed schedule for listening slots. Nihao [12] takes this approach in which there are more transmitting than listening slots in a given period. Furthermore, in Hello [13], nodes listen more at the beginning of the period and periodically wake up for transmissions. This scheme can be considered a generalization of several other mechanisms, such as Disco, U-Connect, and Searchlight.

In contrast, stochastic schemes such as Birthday [14], Aloha-like [15], and others [16,17,18], allow nodes to transmit beacons, listen for beacons from other nodes or sleep based on a probability distribution. In Panda, each sensor remains asleep initially and, then, the node sleeps with the sleep time following an exponential distribution. Following the sleep step, sensors wake up and listen for a constant time. If no packet is received in the listening state, the node broadcasts one packet to others. However, as aforementioned, none of the stochastic algorithms can guarantee successful discovery for the worst-case scenario or provide an upper bound on the discovery latency among the nodes. The only metric applicable is the expected time to discover all neighbors.

As can be noted in Table 1, a plethora of ND protocols has been proposed in the last two decades, with new solutions still appearing even today. Recently, the authors in [34] established a relationship between optimal discovery latency, channel utilization, and duty cycle. According to their analysis, some recent proposals perform optimally and cover parts of the latency/channel utilization/duty-cycle Pareto front. As these authors suggest, the coverage of the entire Pareto front implies no further potential for improvement. However, there is still potential to improve the robustness against non-ideal conditions [34]. Indeed, as far as the authors know, only a few related studies consider performance evaluations under non-ideal conditions. For instance, the authors in [19] assume a certain probability that an unreliable link would affect the discovery. Within this scenario, they proposed both deterministic and stochastic algorithms to solve the asynchronous ND problem with an unreliable link model. In [21], the authors propose Spear, a practical neighbor discovery framework that promises to reduce communication collisions, thus boosting the coincidence rate of existing ND protocols. However, the results of both [19,21] evaluations relied on the choice of protocols, their parametrizations, and the assumed setups. Hence, while a specific protocol might outperform others in such a comparison, it might perform differently if the parametrization or setup is changed. Moreover, for many protocols, it is also not clear how to optimally parametrize them. Given the different kinds of neighbor discovery protocols, there has also been no standard way of comparing them and their performance [34]. A common practice in literature consists of evaluating the performance of most ND protocols using the cumulative distribution function (CDF) of the discovery time and the worst-case boundary. Nevertheless, the worst-case boundary can only exist if there are no transmission errors, that is, under ideal conditions.

Consequently, this paper presents a framework capable of estimating the CDF of deterministic algorithms under non-ideal conditions. In particular, despite the difference in underlying principles (i.e., classifications), deterministic protocols exhibit some similarities. For instance, the active slots in Disco and anchor nodes in Searchlight show a repetitive pattern under both symmetric and asymmetric duty cycles [35]. This key observation makes it possible to develop a framework to model the CDF of deterministic protocols (i.e., Quorum, Disco, U-Connect, Hello, and Searchlight) under non-ideal conditions regardless of their parametric setups.

## 3. Framework to Model Deterministic Algorithms

The CDF for two devices represents the probability that a coincidence between two devices already occurred at a given time. In particular, we considered pairwise discovery since most of the related works assume that nodes join an IoT network gradually, and the discovery procedure takes place only between the nearest neighbors. Moreover, in general, it can be considered that discovering multiple devices always relies on pairwise ND. In particular, we consider the pairwise discovery paradigm since the selected ND protocols develop their respective analytical CDF (i.e., under ideal conditions) based on this particular scenario.

**Figure 1:**
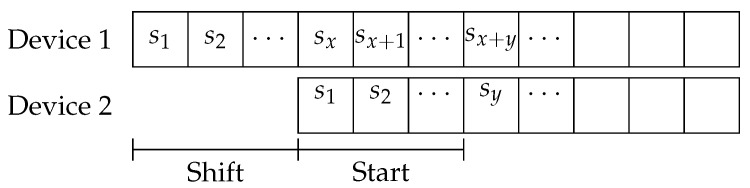
Slot structure used to obtain the CDFs.

The CDF considers all the possible shifts between the two devices regarding the time in which they started to operate and the moment they came in range of each other. Figure 1 shows that the shift is the number of slots between the two devices given that the ND protocol of device two was started after the ND protocol of device one. This value can be arbitrarily large, and is obtained through a random uniform variable. The start slot is a random time that represents the effect of two nodes, already running their protocol, becoming neighbors at an arbitrary moment.

Many physical and MAC layer impairments can disrupt a wireless transmission from the transmitter to the receiver. For instance, channel-induced errors and collisions may prevent the receiver from getting the transmitted packet correctly at the physical layer. At the MAC layer, radios in small sensors are usually half-duplex. As a result, even if nodes wake up simultaneously, a successful reception means one node is transmitting while the other node is in receive mode; any different combination will not work. Even synchronizing nodes such that they wake up simultaneously is troublesome as clocks drift over time. Although it is difficult to consider the impact of one of these error sources in the performance of ND protocols, is far too complex to conduct a performance analysis for all of them simultaneously, at least in a single piece of research.

For this reason, this paper models transmission errors using a simple random variable that could be controlled. While this simplified error model strategy does not model any actual physical or MAC layer error source, it simplifies the analysis required to develop the framework. This simplified error model will can respond to some critical issues, such as the widespread impact of transmission errors on a particular ND protocol or, more importantly, which of the considered ND protocols are more resilient to transmission errors.

For this purpose, let $P_{s}$ be the probability that a transmission is successful. Any $P_{s}$ value below one means that there exist some transmission errors with probability $P_{e}=1-P_{s}$. For a neighbor discovery to be considered successful, it is necessary that node A finds node B and vice versa. Therefore, node A has a probability of success $P_{s}$ to discover node B, and node B has a probability of success $P_{s}$ to discover node A.

Deterministic algorithms behave different from stochastic ones since they ensure the coincidence at most in their corresponding worst case. Nevertheless, when operating under non-ideal conditions, errors might occur that eliminate such boundaries.

**Figure 2:**
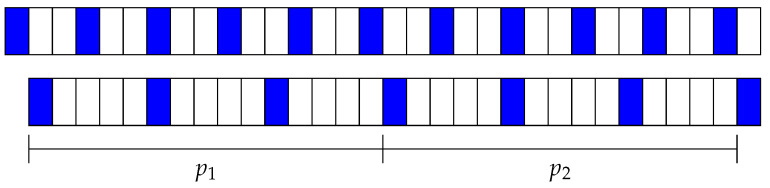
Example of the phases in deterministic neighbor discovery protocols.

To model the CDF of deterministic algorithms in non-ideal conditions, time is divided into phases $p_{n}$. For each algorithm, the phase length is the number of slots required to start a new cycle (i.e., the same pattern of active/inactive slots) between both devices and is denoted as *ℓ*. Figure 2 shows an example of the phases for two asymmetric devices. Device 1 is active for one slot and then inactive for two slots. Device 2 is active for one slot and inactive for four slots. Thus, Device 1 has a 33% duty cycle while Device 2 has a 20% duty cycle. The phase length *ℓ* for those two devices is 15 since the pattern is repeated every 15 slots. For example, phases marked as $p_{1}$ and $p_{2}$ always begin with an inactive slot for Device 1 and an active slot for Device 2; the second slots are always inactive for the two devices, and so on.

For some symmetric protocols (i.e., Hello and U-Connect), *ℓ* might equal the worst-case boundary. It is not the case with Quorum, for which *ℓ* is the entire m×m square. For asymmetric protocols, the value of *ℓ* can be computed as:

(1) $$\ell =pf_{1}\cdot pf_{2},$$

where $pf_{1}$ and $pf_{2}$ are the periodic frame sizes of a protocol for nodes 1 and 2, respectively. Periodicity assumes that the sequence of active/inactive slots will repeat itself after pf slots.

**Figure 3:**
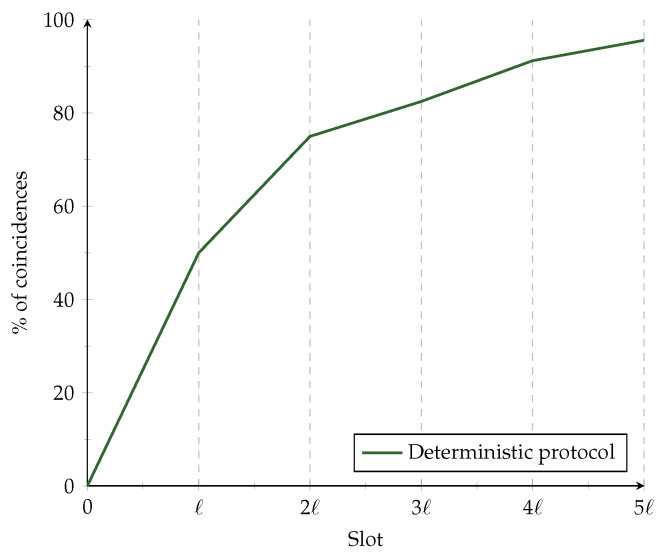
Fixed interval behavior of deterministic algorithms.

When deterministic algorithms are subject to errors, they have a fixed interval behavior, as can be seen in Figure 3. The length of each interval is *ℓ*. During subsequent intervals, the behavior is similar to the previous ones but scaled. Equation (2) represents the CDF composed of two parts: the behavior during the current phase and the coincidences during the former phase.

(2) $$P\left(c\leq n\right)=f\left(n\;\mathrm{mod}\;\ell \right)\cdot \left[P_{fs}\left(p_{n}\right)-P_{fs}\left(p_{n}-1\right)\right]+P_{fs}\left(p_{n}-1\right),$$

where *f* is a function representing the behavior during each phase under ideal conditions and $P_{fs}\left(p_{n}\right)$ is the probability that a coincidence may occur during phase $p_{n}$ given the probability of success $P_{s}$. In this paper, we consider that *f* can be: (i) A straight line from the origin to the worst-case and 100% of coincidences; (ii) The CDF under ideal conditions. In some cases, it is possible to develop functions that represent the protocol’s behavior more accurately by making no assumptions in the ideal CDF.

$p_{n}$ is the current phase and is closely related to the protocol design. For each protocol, $p_{n}$ is computed as:

(3) $$p_{n}=\left\lfloor \frac{n}{\ell }\right\rfloor .$$

**Figure 4:**
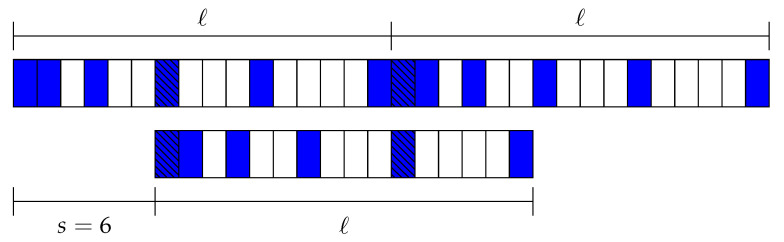
Example for the $c\left(s\right)$ function.

When the protocol is symmetric, $P_{fs}$ can be computed through Equation (4). The equation computes the mean probability that the coincidence may occur in phase $p_{n}$, considering that the coincidence did not occur in previous phases. This computation considers all the possible phase shifts and the number of coincidences in each of those shifts.

(4) $$P_{fs}\left(p_{n}\right)=\left\{\begin{array}{cc} 0 & p_{n}=0\\ \frac{1}{\ell }\cdot \sum_{s=0}^{\ell }1-{\left(1-P_{s}^{2}\right)}^{c(s)}\cdot {\left[{\left(1-P_{s}^{2}\right)}^{c(s)}\right]}^{p_{n}-1} & p_{n}>0, \end{array}\right.$$

where $c\left(s\right)$ is a function representing the number of coincidences that the protocol has in a single phase, given a shift of *s* slots between the two devices, this function needs to be computed by analyzing the behavior of the evaluated protocol. It will be detailed later for each considered protocol as an illustration. Figure 4 shows an example of function $c\left(s\right)$ for a symmetric protocol, where the length of *ℓ* is 16; therefore, the same sequence will be repeated after 16 slots. The active slots are 1, 2, 4, 7, 11, and 16. This figure shows a shift of 6 slots between the starting time of the two devices, so it is used to compute $c\left(6\right)$. For that particular shift, the deterministic protocol will have exactly two coincidences located at slots 1 and 11.

The following subsections present a brief description of four deterministic ND protocols, their ideal CDF, and their non-ideal CDF derivation by applying the proposed framework.

### 3.1. Disco

**Figure 5:**
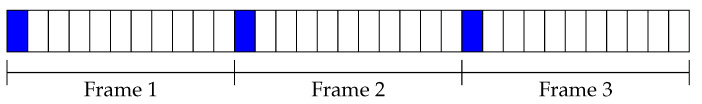
Example of Disco for p=11.

Disco [8] is based on the Chinese Remainder Theorem and uses prime numbers to ensure slot coincidence between devices. It introduces the idea of using relative prime numbers as the number of slots in a frame for a given device. Device *d* will have a frame of size $p_{d}$. Then, each device activates the first slot of the frame, that is, in slots 0, $p_{d}$, $2p_{d}$, and so forth. An example of Disco slot structure for one device is shown in Figure 5 for p=11. Active slots are always located at the beginning of each frame and are colored in blue.

Using different relative prime numbers $p_{1}$ and $p_{2}$ for devices 1 and 2 ensures that Disco has a coincidence in at most $p_{1}\cdot p_{2}$. It is essential to highlight that if $p_{1}$ and $p_{2}$ are not relative primes, the coincidence might not occur. The CDF of Disco in Equation (5) is a straight line from 1 to $p_{1}\cdot p_{2}$, which means that any value has the same probability of occurrence.

(5) $$P\left(c\leq n\right)=\frac{n}{p_{1}\cdot p_{2}}.$$

As Disco uses two different relative prime numbers, it is inherently asymmetric, and to model it, we use Equation (4) with $\ell =p_{1}\cdot p_{2}$. Disco has exactly one coincidence every $p_{1}\cdot p_{2}$ slots. Then, the $c\left(s\right)$ of Disco is represented by Equation (6).

(6) $$c\left(s\right)=1.$$

### 3.2. Quorum

**Figure 6:**
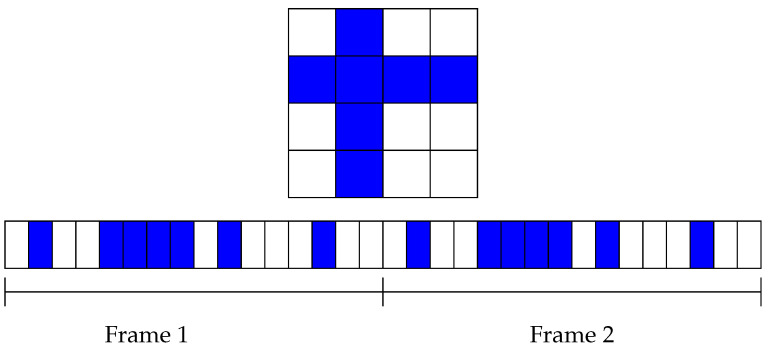
Example of Quorum for m=4.

There are many algorithms based on Quorum techniques. In the one presented in [22], the algorithm uses an m×m matrix as the frame structure. Then, one row and one column from that matrix are chosen for active slots, which ensures that two devices will have at least two coincidences upon a frame even if both devices selected different rows and different columns. Figure 6 shows an example of a 16-slot frame for one device. The upper part shows a matrix representation where the second row and column are selected. The active slots are blue, whereas the inactive slots are white. The bottom of the figure shows the same choice of row and column but is represented as continuous slots over time.

In the worst-case scenario, the devices will have a coincidence at most in $m^{2}$ slots. The cumulative discovery latency of this Quorum system is given by:

(7) $$P\left(c\leq n\right)\approx 1-{\left(1-\frac{n}{m^{2}}\right)}^{2}.$$

Quorum uses symmetric duty cycles, so Quorum performance under non-ideal conditions can be described by Equation (4). First, we can note that Quorum has a phase length of $\ell =m^{2}$, which is different from the worst-case since Quorum ensures at least two coincidences in a phase. To obtain the $c\left(s\right)$ of Quorum, we first make some clarifications. First, in the case of a perfect shift (when the shift is a multiple of *ℓ*), the number of coincidences is 2m−1. Otherwise, when $\left|shift\right|<m$, the number of coincidences is $m+1-\left|shift\right|$ because part of the active slots row has overlapped. Otherwise, when shiftmodm is 0, the number of coincidences is *m*. This case represents an overlap in the active slots column. In any other case, the number of coincidences is 2. Notice that $\left|shift\right|$ represents the relative shift, that is, $\left|shift\right|=\min\left(shift,m^{2}-shift\right)$. Equation (8) summarizes the behavior of $c\left(s\right)$ for Quorum as follows:

(8) $$c\left(s\right)=\left\{\begin{array}{cc} 2m-1 & shift\;\mathrm{mod}\;m^{2}=0\\ m+1-\left|shift\right| & 0<\left|shift\right|<m\\ m & shift\;\mathrm{mod}\;m=0\wedge shift\;\mathrm{mod}\;m^{2}\neq 0\\ 2 & otherwise. \end{array}\right.$$

### 3.3. Hello

**Figure 7:**
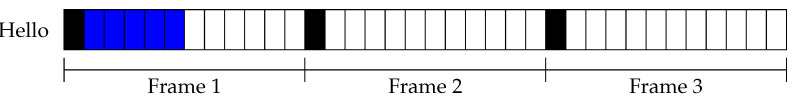
Example of Hello.

Hello [13] is a generalization of U-Connect. It assumes that the frame size might not be a prime number for two nodes with the same duty cycle. Devices will be active at the first slot of each frame and inactive through the rest of the frame. To ensure that a coincidence occurs, device *u* has to be active for the first $\left\lceil \frac{\varsigma_{u}}{2}\right\rceil$ slots every $\varsigma_{u}$ frames. Figure 7 displays an example of the slot structure in one device for ς=12 where the black slots, called *guardians* [13], represent the active slots because those are the first of each frame. Blue slots are active because those are the first $\left\lceil \frac{\varsigma_{u}}{2}\right\rceil$ in the first frame and are referred to as *patrols*. Notice that the blue slots will appear again after ς frames.

In Hello, devices 1 and 2 will have a coincidence at most at $\varsigma_{1}\cdot \varsigma_{2}$ slots. The discovery latency for the case $\varsigma_{1}=\varsigma_{2}=\varsigma$ can be approximated as:

(9) $$P\left(c\leq n\right)\approx \frac{\left\lfloor n/\varsigma \right\rfloor }{\varsigma^{2}}.$$

The symmetric version of Hello relies on devices having the same duty cycle. Equation (4) is used to compute Hello’s behavior under non-ideal conditions. For Hello, $c\left(s\right)$ has four cases according to the protocol: when the two devices are fully synchronized, when the absolute value of the shift between both devices is less than half the frame size ς, when the shift is a modulus of ς, and all the other cases. For Hello, we define the absolute value of the shift as $\left|shift\right|=\min\left(shift,\varsigma^{2}-shift\right)$. Equation (10) models the behavior of $c\left(s\right)$ for Hello in each of the above cases.

(10) $$c\left(s\right)=\left\{\begin{array}{cc} \varsigma +\lfloor \frac{\varsigma }{2}\rfloor & shift\;\mathrm{mod}\;\varsigma^{2}=0\\ \left\lfloor \frac{\varsigma }{2}\right\rfloor +1-\left|shift\right| & 0<\left|shift\right|<\left\lfloor \frac{\varsigma }{2}\right\rfloor \\ \varsigma & shift\;\mathrm{mod}\;\varsigma =0\wedge shift\;\mathrm{mod}\;\varsigma^{2}\neq 0\\ 1 & otherwise. \end{array}\right.$$

In the case of Hello, for function *f*, we will use a line since the CDF has the same form.

### 3.4. Searchlight

Searchlight [10] uses two active slots during each frame, namely an anchor and a probe. The anchor is always the first slot in the frame, and the probe varies from frame to frame. The original manuscript shows that it is sufficient to choose the probe from the first (or last) half of the frame to guarantee an anchor-probe coincidence. Thus, Searchlight ensures that the coincidence occurs at most after $f=\lfloor \frac{t}{2}\rfloor$ frames under ideal conditions, where *t* is the number of slots in a frame.

**Figure 8:**
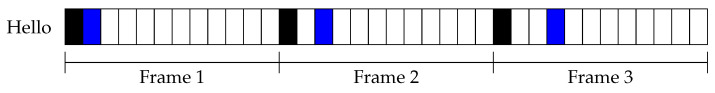
Example of Searchlight-S for t=12.

Although the original manuscript presents two versions, we focused on Searchlight-S (sequential) since Searchlight-R (random) has a stochastic component. Searchlight-S chooses the probe sequentially in a round-robin manner. The example in Figure 8 displays the first three frames of a single device for Searchlight-S when t=12. The black slots are the anchors, and the blue slots represent the probes. It can be seen that anchors are always at the beginning of each frame while the probe sequentially moves to the right. The process will continue up to the $\left\lfloor \frac{t}{2}\right\rfloor$-th frame, after which the probe will restart to the same position as in Frame 1.

In the literature, there is not a latency model for Searchlight-S. However, we propose an approximation as:

(11) $$P\left(c\leq n\right)=\frac{2}{t\cdot \lfloor \frac{t}{2}\rfloor }\cdot n\approx \frac{n}{f^{2}}.$$

For the symmetric Searchlight-S version, we have four cases to compute function $c\left(s\right)$. The first case is when devices are fully synchronized, so all possible anchors and probes are coincidences (there are *t* coincidences). The second case is when the shift is equivalent to a phase, all the anchors are coincidences, but no probe will coincide. The third case is when the shift between devices is the largest possible, that is, $\left\lfloor \frac{t}{2}\right\rfloor$. In that case, one probe from each device has a coincidence with an anchor. In the last case, the devices tend to synchronize their probes. This way, a fraction of the probes coincide while there is only one anchor-probe coincidence. In any other case, there is one coincidence. The function that summarizes the five cases is presented in Equation (12).

(12) $$c\left(s\right)=\left\{\begin{array}{cc} t & shift\;\mathrm{mod}\;t\lfloor \frac{t}{2}\rfloor =0\\ \left\lfloor \frac{t}{2}\right\rfloor & shift\;\mathrm{mod}\;t=0\wedge shift\;\mathrm{mod}\;t\lfloor \frac{t}{2}\rfloor \neq 0\\ 2 & shift\;\mathrm{mod}\;t=\lfloor \frac{t}{2}\rfloor \\ \lfloor \frac{t}{2}\rfloor -i+1 & shift=\left(i\cdot t+i\right)\forall 0<i<\left\lfloor \frac{t}{2}\right\rfloor \wedge i\in Z\\ \lfloor \frac{t}{2}\rfloor -i+1 & t\left\lfloor \frac{t}{2}\right\rfloor -shift=\left(i\cdot t+i\right)\forall 0<i<\left\lfloor \frac{t}{2}\right\rfloor \wedge i\in Z\\ 1 & otherwise. \end{array}\right.$$

### 3.5. Stochastic Algorithms

Stochastic algorithms are inherently unbounded since it is impossible to predict a limit upon which the coincidence is guaranteed. Stochastic trials are good in practice despite the lack of boundaries since they have a lower mean coincidence time. Although the presented framework cannot be applied to these protocols, they were considered to compare their performance under unreliable transmissions.

#### 3.5.1. Birthday

In [14], the authors propose a neighbor discovery technique based on the birthday math problem where the probability of encounter between devices increases when the number of nodes increases. In Birthday BLT, the devices may appear in three states: sleep, transmit or receive. In each slot, a device will choose its state randomly. It will be in transmit state with a probability of $p_{t}$, receive state with a probability of $p_{r}$, and sleep with a probability of $1-p_{t}-p_{r}$. Since it is a stochastic technique, it cannot ensure that the coincidence will occur after a given time. On the other hand, Birthday’s discovery latency can be expressed as:

(13) $$P\left(c\leq n\right)=1-{\left(1-p_{t}\cdot p_{r}\right)}^{n}.$$

Equation (14) models Birthday’s CDF under non-ideal conditions, as follows:

(14) $$P\left(c\leq n\right)=1-{\left[1-2\cdot \left(P_{s}\cdot p_{t}\right)\cdot \left(P_{s}\cdot p_{r}\right)\right]}^{n}.$$

When $p_{t}=p_{r}=\frac{p}{2}$, Equation (14) becomes Equation (15). Values of $p_{t}$ and $p_{s}$ are chosen to maintain a duty cycle of *p* and have the same transmitting or receiving probability.

(15) $$P\left(c\leq n\right)=1-{\left[1-\frac{{\left(P_{s}\cdot p\right)}^{2}}{2}\right]}^{n}.$$

#### 3.5.2. Random

Optimization of the basic Birthday protocol refers to the process in which each device randomly chooses its active slots according to its duty cycle. So, when two devices coincide at the same active slot, they exchange information and identify as neighbors. For simplicity, throughout this paper, we assume that devices send beacons at the beginning and end of each active time slot and listen to beacons from other devices during the remaining slot time. Although this is not the only strategy designed to exchange information between nodes in an active slot, it is by far the most widely used strategy in the literature [19].

Let *p* be the node’s duty cycle. That is, the probability that a node is active in a particular slot is also *p*. We define the probability that a couple of devices are simultaneously active, for the first time, at slot *n* as:

(16) $$P\left(c=n\right)={\left(1-p^{2}\right)}^{n-1}\left(p^{2}\right).$$

So, the discovery latency in the Random protocol can be expressed as:

(17) $$P\left(c\leq n\right)=\sum_{i=1}^{n}{\left(1-p^{2}\right)}^{i-1}\left(p^{2}\right).$$

Then, the probability that a coincidence occurred at most at the *n*-th slot is represented as:

(18) $$P\left(c\leq n\right)=1-{\left(1-p^{2}\right)}^{n}.$$

Plain [19] is one of the few protocols designed to work under unreliable scenarios. Its behavior, as well as its CDF, are very similar to those of Random. The main difference is that Plain will always be active in $\frac{p}{100}$ slots, while Random might not always accomplish this (although in average it does accomplish it). For this reason, this paper only considers Random.

The Random protocol is extended to non-ideal conditions by adding the probability of success $P_{s}$ to Equation (17) as follows:

(19) $$P\left(c\leq n\right)=\sum_{i=1}^{n}{\left(1-{\left(P_{s}\cdot p\right)}^{2}\right)}^{i-1}\left({\left(P_{s}\cdot p\right)}^{2}\right)$$

(20) $$P\left(c\leq n\right)=1-{\left(1-{\left(P_{s}\cdot p\right)}^{2}\right)}^{n}.$$

## 4. Framework Validation

This section validates the proposed framework using simulations developed in Python. For this, we implemented the behavior of each protocol according to their specifications, adding the probability of success $P_{s}$. Each protocol was simulated 100k times using a different seed for each experiment. The seed changed random numbers, shifts, and the start of each simulation for each protocol. A coincidence occurs when the protocol dictates that both devices are active during the same slot and a uniform randomly generated number $R\leq P_{s}$ for each of the two devices. The simulation parameters for each protocol for 10% and 1% duty cycle are shown in Table 2. Those parameters are selected to accomplish the required duty cycle as close as possible.

### 4.1. Simulator Validation under Ideal Conditions

**Figure 9:**
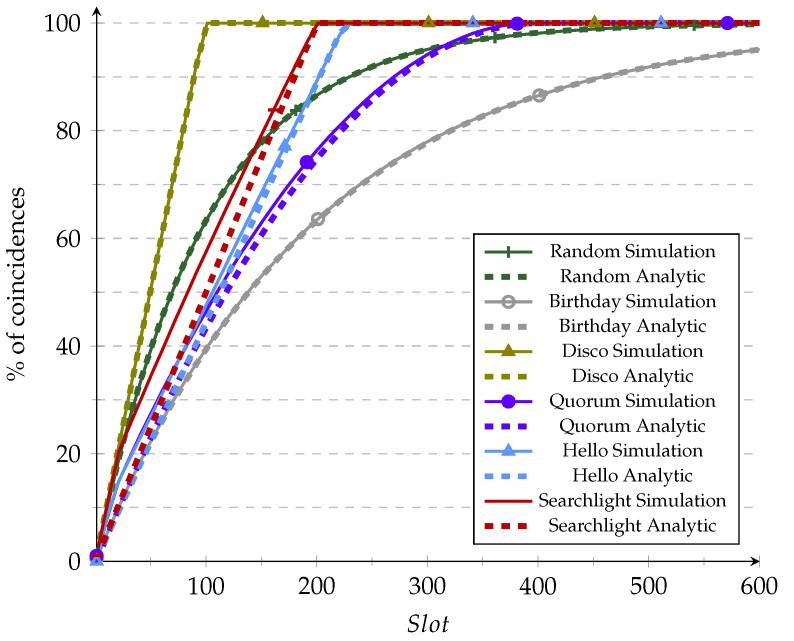
Validation of the simulation vs. analytic models for 10% duty cycle.

**Figure 10:**
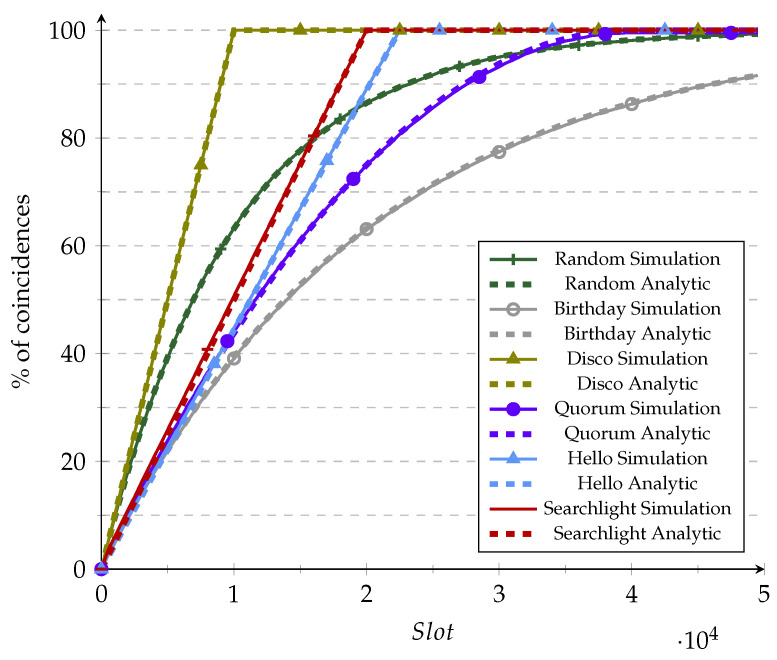
Validation of the simulation vs. analytic models for 1% duty cycle.

We used Figure 9 and Figure 10 to validate the simulator accuracy versus the analytical CDF already found in the literature under ideal conditions (i.e., $P_{s}=1$) before moving to perform extensive simulations in non-ideal conditions. This validation was performed under ideal conditions since those are the only CDFs available in the literature for the reviewed protocols. It should be recalled that some analytic CDFs relied on assumptions regarding the shifts to obtain a more straightforward equation. Usually, the obtained CDF does not consider the cases in which the shift is perfect or a modulus of the period [7,22]. When frame size increases, these cases are less likely to occur, and thus the approximation becomes more accurate. This effect can be observed in Figure 10, in which the analytic plots fit much better than in Figure 9, in which the duty cycle is higher (and the frame size is consequently smaller).

### 4.2. Analytic Model Validation under Non-Ideal Conditions

The model proposed in Section 3 was validated via simulations for each protocol. We present comparisons between analytic and simulation plots and the error between both to characterize the discrepancies. Simulations were run 100k times to maximize the convergence of the protocol and decrease the error due to simulations. Each of the simulations uses a randomly generated *shift* and *start* to comply with CDF definitions. There are two devices in each simulation, and we plotted the protocol’s analytical CDF, simulations, and the error between both.

**Figure 11:**
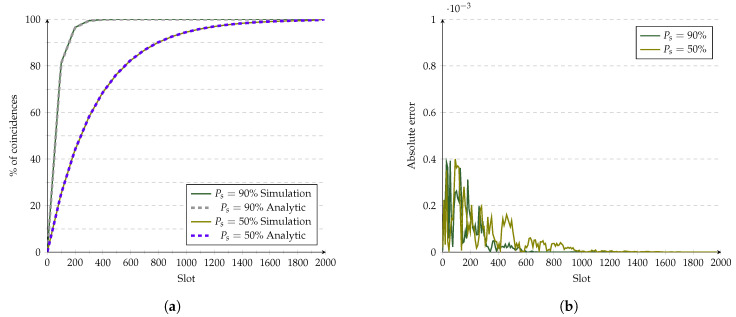
Evaluation of Disco’s analytic model at 10% duty cycle. (**a**) Analytic model vs. simulations. (**b**) Error of the analytic model.

Figure 11 shows the validation for Disco. Its CDF under ideal conditions behaves quite linearly, and $f\left(n\;\mathrm{mod}\;\ell \right)$ is also modeled as a line with $f\left(x\right)=\frac{x}{p_{1}p_{2}}$. Accordingly, in Figure 11a, the approximation of the analytic model fits very well compared with the simulations. The error plotted in Figure 11b never goes over $0.4\times 10^{-3}$. It is thus related to the randomness of the simulations. To prove this, we ran the experiments ten more times, and the mean error in the interval $\left(0,2000\right]$ decreased three times compared with 100k simulations. However, the time needed to complete 1 million experiments took ten times longer.

**Figure 12:**
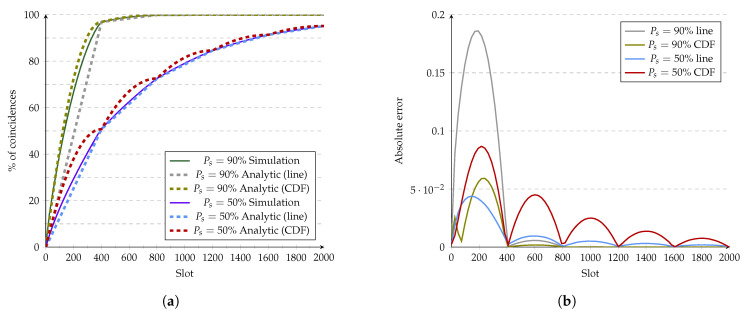
Evaluation of Quorum’s analytic model at 10% duty cycle. (**a**) Analytic model vs. simulations. (**b**) Error of the analytic model.

For Quorum, we use two different approaches of $f\left(n\;\mathrm{mod}\;\ell \right)$, as shown in Figure 12. The first approach uses *f* as a straight line with $f\left(x\right)=\frac{x}{m^{2}}$, while the second approach uses the Quorum’s CDF under ideal conditions $f\left(x\right)=1-{\left(1-\frac{x}{m^{2}}\right)}^{2}$. Figure 12a, shows that none of the models fit perfectly, like in Disco. Instead, both approaches demonstrate different behaviors. The straight-line approach tends to be under the simulations line, while the CDF approach is above for both $P_{s}=50\%$ and $P_{s}=90\%$. On the other hand, Figure 12b shows that the CDF overcomes the line approximation for $P_{s}=90\%$ in the first phase. Nevertheless, for $P_{s}=50\%$, the line approximation produced better results. These plots show that the proposed model’s accuracy is closely related to selecting $f\left(n\;\mathrm{mod}\;\ell \right)$.

**Figure 13:**
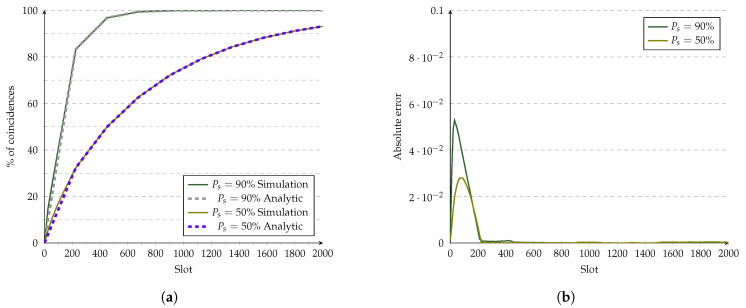
Evaluation of Hello’s analytic model at 10% duty cycle. (**a**) Analytic model vs. simulations. (**b**) Error of the analytic model.

An ideal CDF of the Hello protocol is modeled as a line. Although authors in [7] state that this is only an approximation and does not consider special cases occuring at the beginning and end of a phase. For that reason, for Figure 13, we modeled $f\left(n\;\mathrm{mod}\;\ell \right)$ as the line represented by $f\left(x\right)=\frac{x}{\varsigma^{2}}$ (in this case, the CDF). Figure 13a shows that the model is not very accurate during the first phase but improves afterwards. The same behavior occurs in Figure 13b, where the maximum error is about 5% for $P_{s}=90\%$ and then decreases until it becomes imperceptible during the third phase and so on. The assumptions of the CDF caused this effect. The ideal CDF did not consider the case where the absolute value of the shift between two devices is lower than $\left\lfloor \frac{\varsigma }{2}\right\rfloor$, where the number of overlapping active slots is more than one. It also did not consider cases where the shift is a multiple of ς, where the number of overlapping active slots in a phase is always ς. Such cases with a high probability of discovery during the first phases are less likely to occur during later phases. Thus, they have much weight only during the first phases and become less significant in later phases.

**Figure 14:**
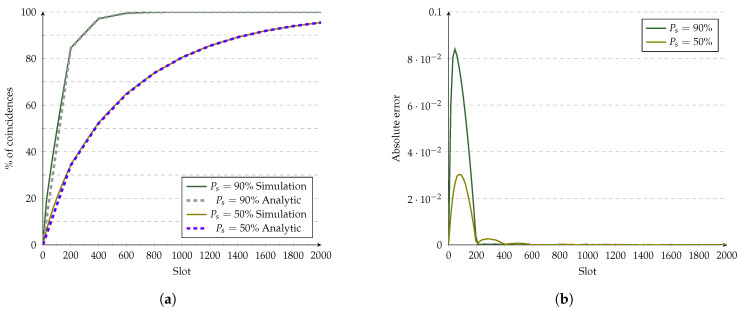
Evaluation of Searchlight’s analytic model at 10% duty cycle. (**a**) Analytic model vs. simulations. (**b**) Error of the analytic model.

Searchlight was evaluated using the ideal CDF as a straight line with $f\left(x\right)=\frac{x}{t\lfloor \frac{t}{2}\rfloor }$. The results are shown in Figure 14. The CDF plotted in Figure 14a shows that the error from Figure 14b has a similar trend to the one observed in Hello (i.e., showing errors that decrease after each phase). This behavior is related to: (i) devices with a perfect shift, the only shift where all the anchors and probes coincide, and (ii) devices with shifts where some probes show coincidences.

**Figure 15:**
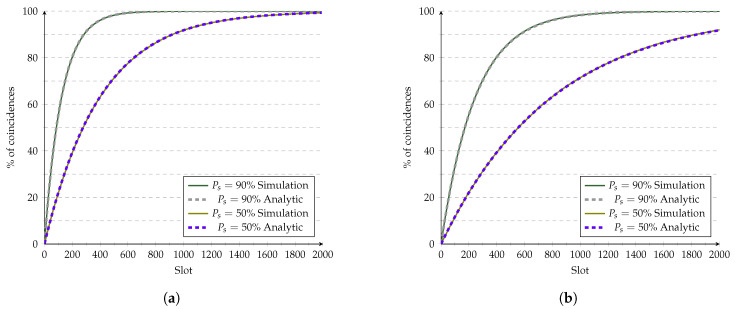
Evaluation of (**a**) Random’s and (**b**) Birthday’s analytic models vs. simulations at 10% duty cycle.

Finally, Figure 15 shows the validations for Random and Birthday protocols, respectively. Both algorithms use uniformly distributed random variables to select the state in a given slot. Furthermore, both use the same approximation to compute their non-ideal CDFs. Both figures show the CDF plots for $P_{s}=90\%$ and $P_{s}=50\%$. Figure 15 show that the obtained approximation fits very well for both probabilities of success.

## 5. Protocol Comparison under Non-Ideal Conditions

When subject to unreliable conditions, neighbor protocols demonstrated a very different behavior from that observed under ideal conditions. This section aims to compare the behavior demonstrated by the six analyzed protocols. Simulations in this section made it possible to handle parameters like duty cycle and probability of coincidence to compare the performance of the neighbor discovery protocols performance under reliable and unreliable conditions. We omit the Plain [19] protocol since the behavior is very similar to that of Random, and the differences are imperceptible.

### 5.1. Duty Cycle vs. Error

**Figure 16:**
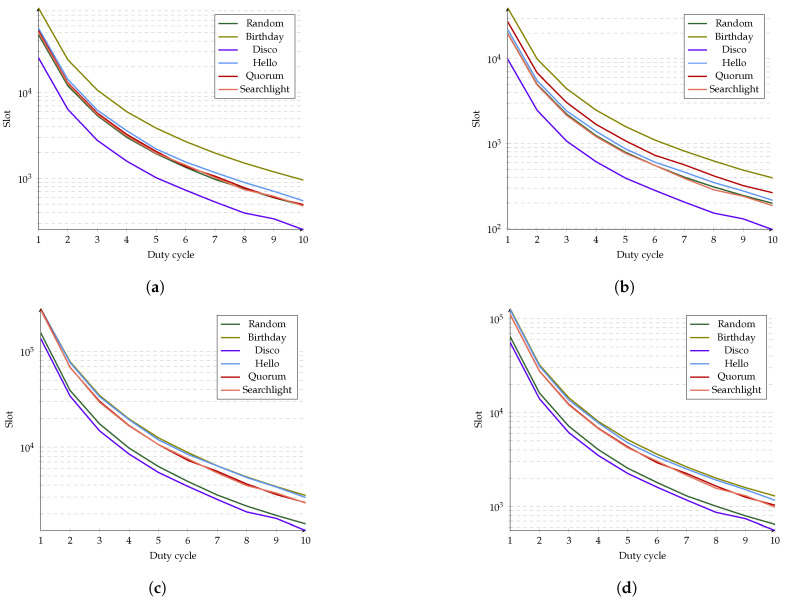
Slot where a percentage of discoveries occur given a duty cycle. (**a**) Ninety-eight percent discoveries, 10% error. (**b**) Eighty percent discoveries, 10% error. (**c**) Ninety-eight percent discoveries, 50% error. (**d**) Eighty percent discoveries, 50% error.

To assess the impact of the duty cycle, we evaluate $P_{s}$ for 90% and 50%. Figure 16 presents plots in which 98% and 80% of coincidences have already occurred in a given slot. Notice that for each plot, when a given algorithm is the *n*-th best algorithm for the given probability of error and encounter probability, it remains the *n*-th best algorithm regardless of the duty cycle. There are minor deviations from this behavior caused by the inaccuracy of the duty cycle.

Figure 16a shows that most protocols reached 98% of coincidences simultaneously, but Disco and Birthday were always the best and worst protocols, respectively. On the other hand, in Figure 16b, there is a more significant difference for 80% of discoveries between Random, Hello, Quorum and Searchlight. In this plot, Disco and Birthday are also the best and the worst, respectively. When evaluated at $P_{s}=50\%$, Figure 16c,d indicate that Birthday, Hello, Quorum and Searchlight have similar behaviors. However, Random and Disco differ from the rest of the protocols and have the best performance for both 98% and 80% of discoveries. It is worth noting that for $P_{s}=50\%$, Birthday’s performance was very similar to that of Quorum, Searchlight, and Hello.

In general, plots indicate that as the duty cycle increases, the number of slots to achieve a discovery decreases for any percentage of coincidence, and error probability ($P_{e}=1-P_{s}$) behavior demonstrates the same trend. This is a natural consequence of each protocol design. In order to change the duty cycle in some protocols, the frame size could be constant, but the number of active slots increases. Other designs have fixed active slots, so frame size decreases.

Comparing Figure 16a,d emphasizes how stochastic protocols perform much better than the rest of the previously described protocols when $P_{s}$ decreases; despite duty cycle changes, they keep overcoming deterministic protocols.

### 5.2. Probability of Coincidence vs. Error

**Figure 17:**
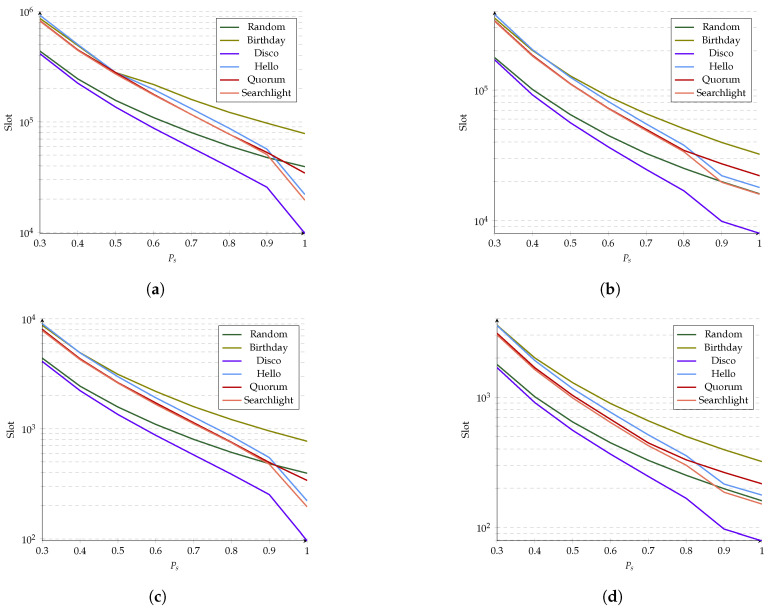
Slots required to achieve a percentage of discoveries given a probability of success. (**a**) Ninety-eight percent of coincidences with 1% duty cycle. (**b**) Eighty percent of coincidences with 1% duty cycle. (**c**) Ninety-eight percent of coincidences with 10% duty cycle. (**d**) Eighty percent of coincidences with 10% duty cycle.

A parameter that affects the relative effectiveness of a particular algorithm compared to others is the probability of success $P_{s}$. As seen in Figure 17, algorithms that have a good performance when $P_{s}=1$ decrease their performance along with $P_{s}$. For example, in Figure 17a, Hello had one of the lowest latencies for $P_{s}=1$ but had the highest latency when $P_{s}<0.5$. This effect is more visible for lower duty cycles and a higher percentage of discoveries. Another example in Figure 17c is the Random algorithm, the second-worst when $P_{s}=1$ but becomes the second-best when $P_{s}<0.85$.

The slope of the plots in Figure 17 provides a good view regarding the sensitivity of each protocol to changes in $P_{s}$. Deterministic protocols have more negative slopes in the presence of transmission errors, which makes them more sensitive to changes in $P_{s}$. On the other hand, stochastic protocols are more resilient to variations in $P_{s}$, and their slopes are less negative. Protocols that have a good performance at achieving 80% of discoveries do not necessarily have good performance for 98% of discoveries. Disco is the only deterministic algorithm that outperforms the stochastic ones in each scenario. This occurs because of the constant value of the $c\left(s\right)$ and the reduced worst-case boundary, allowing all possible shifts to have the same probability of coincidence. Moreover, the other deterministic protocols reviewed benefited some shifts but harmed others while having greater worst-case boundaries.

## 6. Discussion

In the literature, most of the proposed ND protocols were designed and tested under ideal conditions. They assumed that whenever two nodes become active in the same slot, neighbor discovery was guaranteed. However, a more realistic scenario would consider channel errors, lack of energy, sync issues, among other factors. All these factors may cause the neighbor discovery process to fail. This paper introduced a novel framework for evaluating the performance of deterministic ND protocols under non-ideal conditions, a framework later validated by simulations. A discussion of the findings is as follows:

In general, reducing the probability of a successful transmission ($P_{s}$) increases the latency for all ND protocols. Nevertheless, the latency of studied ND protocols is not likewise affected by the probability of error $P_{e}$ (recall that $P_{e}=1-P_{s}$). Table 3 summarizes results from Figure 17, showing the number of slots required to achieve 90% and 98% of coincidences under several values of error probability (i.e., 0%, 30%, and 50%). As can be noted, a $P_{e}$ of 30% doubles the number of slots required for stochastic algorithms to reach 90% and 98% of coincidences compared with a $P_{e}$ = 0%. In contrast, deterministic protocols require 2.27 and 5.78 times more slots to reach 90% and 98% of coincidences for a $P_{e}$ = 30% versus a $P_{e}$ = 0%. Furthermore, for $P_{e}$ = 50%, while stochastic protocols require four times more slots than for $P_{e}=0\%$, deterministic protocols require up to 14.04 times more slots under similar conditions. The above results show that deterministic ND protocols perform well but only under ideal conditions. Once transmission errors appear, these protocols’ rigid, precise operation breaks, requiring more slots to discover a neighbor than otherwise simple, memory-less stochastic protocols.

In most deterministic protocols, if the frame shift between two nodes is known, the slot in which the two nodes will discover each other under ideal conditions can almost be predicted. This operation predicts in which slots the two nodes will meet and in which other slots they will not meet. Together with the periodic behavior of deterministic protocols, this condition made it possible to design the framework presented in this paper. This framework requires computing three main parameters from the protocol operation, namely, the period length *ℓ*, the function describing the coincidences given a shift $c\left(s\right)$, and the function describing the protocol’s behavior during each phase $f\left(n\;\mathrm{mod}\;\ell \right)$. The presented framework can save much time in evaluating the performance of a newly designed deterministic ND protocol since it takes much less time to obtain results compared to simulations.

The *ℓ* and $c\left(s\right)$ can be directly obtained for each protocol specification. On the other hand, $f\left(n\;\mathrm{mod}\;\ell \right)$ needs to be computed not necessarily from the protocol specification. To obtain quick results, $f\left(n\;\mathrm{mod}\;\ell \right)$ can be easily approximated as a straight line from the origin to the worst-case boundary (and 100% of the coincidences). This approximation will have better results for low $P_{s}$ values, even if the ideal CDF is unknown. Besides, the selection of $f\left(n\;\mathrm{mod}\;\ell \right)$ for a protocol is crucial to the framework’s performance. A poor function choice might lead to greater errors, especially for high values of $P_{s}$.

The fact that some active slots in deterministic protocols have a different probability of discovery plays against these protocols once transmission errors occur. Consider, for example, the slot where the protocol’s operation predicted a discovery under ideal conditions, but failed due to transmission error. In this case, it will take many more active slots for both nodes to again reach a slot in which discovery becomes feasible. On the other hand, stochastic protocols have approximately the same probability of discovery in each active slot. If two nodes miss a discovery in one active slot due to transmission errors, they will have the same probability of discovery in the following active slot. The later behavior better suits operation under non-ideal conditions, and this is the reason behind the lower increase in latency values for the Random and Birthday protocols found in Table 3.

It should be recalled that the main goal behind most ND protocols is to discover neighbor nodes with the lowest possible bounded latency. The pursuit of this goal has led to an underestimation of simple stochastic protocols that do not provide such bounds and can lead to potentially long delays. The results presented in this work should lead to a reconsideration of this paradigm. As this work demonstrates, bounded delays for deterministic algorithms are no longer feasible in the presence of transmission errors. Their main advantage over stochastic algorithms thus disappears. This consideration should encourage a departure from traditional bounded delays and CDF metrics and should make it possible to consider other metrics for protocol evaluation in the future, such as the expected latency. The design process of future ND protocols must consider the impact of transmission errors from the start and not only as part of their performance evaluation.

Finally, this work did not consider power consumption in the overall evaluation of the protocols. While it seems apparent that longer latency values due to transmission errors will translate into higher power consumption, it is a topic that needs to be addressed carefully, especially now that bounded delays are no longer present. While this paper summarizes all the non-ideal conditions that may give rise to transmission errors, it might happen that the discovery failed simply because one node did not have enough energy to transmit in energy-harvesting scenarios. In this latter case, the discovery failure did not account for any power consumed by the node. This and other power-related issues need to be considered in the future.

## 7. Conclusions

Neighbor discovery remains a crucial task for the adequate operation of many IoT applications, allowing wireless-enabled nodes to find each other in power-depleted environments. While previous studies of ND protocols considered ideal communication conditions, this paper proposes a new framework for evaluating the performance of deterministic neighbor discovery protocols when transmission errors are present. This framework can be used even if the error-free CDF of the ND protocol is unknown and can yet achieve lower errors. In particular, the proposed framework characterizes an ND protocol in terms of its fixed-length interval behavior given the set of possible *shifts*. The proposed framework was applied to four of the most representative deterministic ND protocols found in the literature (i.e., Disco, Searchlight, Quorum, and Hello) to illustrate its usage and validate the framework’s accuracy. Comparing the behavior of the four selected protocols, as well as two stochastic ND protocols (i.e., Birthday and Random) which were added for comparison purposes, we found, in general, neighbor discovery latency increases for all the considered protocols as the probability of transmission failure increases. However, results suggest that deterministic ND protocols are more vulnerable to performance degradation than non-deterministic ND protocols that are more resilient to transmission errors. This framework shortens the time needed to study the impact of transmission errors on existing and future designs of ND protocols that will otherwise have to be implemented in a simulator, a process that can be time-consuming and is prone to error.

## Figures and Tables

**Table 1:** Summary of the most significant ND protocols in the literature.

Year	Deterministic				Stochastic
	Quorum-Based	Prime Number-Based	Dynamic Listen Slot	Fixed Listen Slot	
1985	Grid [2]				
1997	Cyclic [4]				
1998	Torus [3]				
2001					Birthday [14]
2003	Quorum [22]				
2005	e-torus [23]				
2008	f-torus [24]	Disco [8]			
2009					Aloha-like [16]
2010	[5]	U-Connect [7]			
2011					[17,25]
2012			Searchlight [10]		Aloha-like [15]
2014		[9]	Blinddate [11]	Hello [13]	[18]
2015	Code-base [6,26]	Todis [27]	Hedis [27]		PSBA [28]
2016				Nihao [12], Q-Connect [29]	Panda [30]
2018					Panacea [31], Alano [32]
2020			PWEND [33]		

**Table 2:** Simulation parameters for 10% and 1% duty cycle.

Duty Cycle	10%	1%
Random	p=0.1	p=0.01
Birthday	$p_{t}=0.05$, $p_{r}=0.05$	$p_{t}=0.005$, $p_{r}=0.005$
Disco	$p_{1}=9$, $p_{2}=11$	$p_{1}=99$, $p_{2}=101$
Quorum	m=20	m=200
Hello	ς=15	ς=150
Searchlight	t=20	t=200

**Table 3:** Slots required to reach 90% and 98% of coincidences for 10% Duty Cycle.

*% of Coincidences*		Random	Birthday	Disco	Quorum	Hello	Searchlight
90%	$P_{e}=0\%$	230	460	89	270	205	175
	$P_{e}=30\%$	475	960	350	613	760	637
		**[2.06]**	**[2.08]**	**[3.93]**	**[2.27]**	**[3.7]**	**[3.64]**
	$P_{e}=50\%$	921	1831	795	1420	1710	1468
		**[4]**	**[3.98]**	**[8.93]**	**[5.25]**	**[8.34]**	**[8.38]**
98%	$P_{e}=0\%$	394	770	96	221	339	195
	$P_{e}=30\%$	801	1589	579	1278	1136	1110
		**[2.03]**	**[2.06]**	**[6.03]**	**[5.78]**	**[3.35]**	**[5.69]**
	$P_{e}=50\%$	1577	3126	1348	2977	2626	2603
		**[4]**	**[4.05]**	**[14.04]**	**[13.47]**	**[7.74]**	**[13.34]**

Results in brackets represent the proportion of slots compared to $P_{e}=0\%$.

## Data Availability

The data presented in this study are available on request from the corresponding author.

## Abbreviations

The following abbreviations are used in this manuscript:

ND	Neighbor Discovery
IoT	Internet of Things
CDF	Cummulative Distribution Function
MAC	Medium Access Control

## References

[1] Ye W., Heidemann J., Estrin D. An energy-efficient MAC protocol for wireless sensor networks. Proceedings of the Twenty-First Annual Joint Conference of the IEEE Computer and Communications Societies.

[2] Maekawa M. (1985). A $\sqrt{N}$ algorithm for mutual exclusion in decentralized systems. ACM Trans. Comput. Syst. (TOCS).

[3] Lang S.D., Mao L.J. A comparison of two torus-based k-coteries. Proceedings of the 10th International Conference on Parallel and Distributed Computing and Systems.

[4] Luk W.S., Wong T.T. Two new quorum based algorithms for distributed mutual exclusion. Proceedings of the 17th International Conference on Distributed Computing Systems.

[5] Lai S., Ravindran B., Cho H. (2010). Heterogenous quorum-based wake-up scheduling in wireless sensor networks. IEEE Trans. Comput..

[6] Meng T., Wu F., Chen G. (2015). Code-based neighbor discovery protocols in mobile wireless networks. IEEE/Acm Trans. Netw..

[7] Kandhalu A., Lakshmanan K., Rajkumar R. U-connect: A low-latency energy-efficient asynchronous neighbor discovery protocol. Proceedings of the 9th ACM/IEEE International Conference on Information Processing in Sensor Networks.

[8] Dutta P., Culler D. Practical asynchronous neighbor discovery and rendezvous for mobile sensing applications. Proceedings of the 6th ACM Conference on Embedded Network Sensor Systems.

[9] Meng T., Wu F., Chen G. On designing neighbor discovery protocols: A code-based approach. Proceedings of the IEEE INFOCOM 2014-IEEE Conference on Computer Communications.

[10] Bakht M., Trower M., Kravets R.H. Searchlight: Won’t you be my neighbor?. Proceedings of the 18th Annual International Conference on Mobile Computing and Networking.

[11] Wang K., Mao X., Liu Y. (2014). BlindDate: A neighbor discovery protocol. IEEE Trans. Parallel Distrib. Syst..

[12] Qiu Y., Li S., Xu X., Li Z. Talk more listen less: Energy-efficient neighbor discovery in wireless sensor networks. Proceedings of the IEEE INFOCOM 2016-The 35th Annual IEEE International Conference on Computer Communications.

[13] Sun W., Yang Z., Wang K., Liu Y. Hello: A generic flexible protocol for neighbor discovery. Proceedings of the IEEE INFOCOM 2014-IEEE Conference on Computer Communications.

[14] McGlynn M.J., Borbash S.A. Birthday protocols for low energy deployment and flexible neighbor discovery in ad hoc wireless networks. Proceedings of the 2nd ACM International Symposium on Mobile ad Hoc Networking & Computing.

[15] Sun G., Wu F., Chen G. Neighbor discovery in low-duty-cycle wireless sensor networks with multipacket reception. Proceedings of the 2012 IEEE 18th International Conference on Parallel and Distributed Systems.

[16] Vasudevan S., Towsley D., Goeckel D., Khalili R. Neighbor discovery in wireless networks and the coupon collector’s problem. Proceedings of the 15th Annual International Conference on Mobile Computing and Networking.

[17] Zeng W., Vasudevan S., Chen X., Wang B., Russell A., Wei W. Neighbor discovery in wireless networks with multipacket reception. Proceedings of the Twelfth ACM International Symposium on Mobile Ad Hoc Networking and Computing.

[18] Song T., Park H., Pack S. A probabilistic neighbor discovery algorithm in wireless ad hoc networks. Proceedings of the 2014 IEEE 79th Vehicular Technology Conference (VTC Spring).

[19] Li W., Zhang J., Jiang F., Li Z., Xu C. (2019). Asynchronous neighbor discovery with unreliable link in wireless mobile networks. Peer -Peer Netw. Appl..

[20] Geissdoerfer K., Zimmerling M. Bootstrapping Battery-Free Wireless Networks: Efficient Neighbor Discovery and Synchronization in the Face of Intermittency. Proceedings of the 18th USENIX Symposium on Networked Systems Design and Implementation.

[21] Gu Z., Wang Y., Shi W., Tian Z., Ren K., Lau F. (2019). A practical neighbor discovery framework for wireless sensor networks. Sensors.

[22] Tseng Y.C., Hsu C.S., Hsieh T.Y. (2003). Power-saving protocols for IEEE 802.11-based multi-hop ad hoc networks. Comput. Netw..

[23] Jiang J.R., Tseng Y., Hsu C.S., Lai T.H. (2005). Quorum-based asynchronous power-saving protocols for IEEE 802.11 ad hoc networks. Mob. Netw. Appl..

[24] Jiang J.R. (2008). Expected quorum overlap sizes of quorum systems for asynchronous power-saving in mobile ad hoc networks. Comput. Netw..

[25] You L., Yuan Z., Yang P., Chen G. ALOHA-like neighbor discovery in low-duty-cycle wireless sensor networks. Proceedings of the 2011 IEEE Wireless Communications and Networking Conference.

[26] Own C.M., Meng Z., Liu K. (2015). Handling neighbor discovery and rendezvous consistency with weighted quorum-based approach. Sensors.

[27] Chen L., Fan R., Bian K., Gerla M., Wang T., Li X. On heterogeneous neighbor discovery in wireless sensor networks. Proceedings of the 2015 IEEE Conference on Computer Communications (INFOCOM).

[28] Chen L., Li Y., Chen Y., Liu K., Zhang J., Cheng Y., You H., Luo Q. (2015). Prime-set-based neighbour discovery algorithm for low duty-cycle dynamic WSNs. Electron. Lett..

[29] Chen H., Lou W., Wang Z., Xia F. (2016). On achieving asynchronous energy-efficient neighbor discovery for mobile sensor networks. IEEE Trans. Emerg. Top. Comput..

[30] Margolies R., Grebla G., Chen T., Rubenstein D., Zussman G. (2016). Panda: Neighbor discovery on a power harvesting budget. IEEE J. Sel. Areas Commun..

[31] Cao Z., Gu Z., Wang Y., Cui H. Panacea: A low-latency, energy-efficient neighbor discovery protocol for wireless sensor networks. Proceedings of the 2018 IEEE Wireless Communications and Networking Conference (WCNC).

[32] Shen T., Wang Y., Gu Z., Li D., Cao Z., Cui H., Lau F.C. Alano: An Efficient Neighbor Discovery Algorithm in an Energy-Restricted Large-Scale Network. Proceedings of the 2018 IEEE 15th International Conference on Mobile Ad Hoc and Sensor Systems (MASS).

[33] Chen H., Qin Y., Lin K., Luan Y., Wang Z., Yu J., Li Y. (2020). PWEND: Proactive wakeup based energy-efficient neighbor discovery for mobile sensor networks. Ad. Hoc. Netw..

[34] Kindt P.H., Chakraborty S. (2021). Performance Limits of Neighbor Discovery in Wireless Networks. arXiv.

[35] Sun W., Yang Z., Zhang X., Liu Y. (2014). Energy-efficient neighbor discovery in mobile ad hoc and wireless sensor networks: A survey. IEEE Commun. Surv. Tutor..

